# DNA Methylation-Based Panel Predicts Survival of Patients With Clear Cell Renal Cell Carcinoma and Its Correlations With Genomic Metrics and Tumor Immune Cell Infiltration

**DOI:** 10.3389/fcell.2020.572628

**Published:** 2020-10-15

**Authors:** Xiao-Ping Liu, Lingao Ju, Chen Chen, Tongzu Liu, Sheng Li, Xinghuan Wang

**Affiliations:** ^1^Department of Urology, Zhongnan Hospital of Wuhan University, Wuhan, China; ^2^Department of Biological Repositories, Zhongnan Hospital of Wuhan University, Wuhan, China; ^3^Human Genetics Resource Preservation Center of Wuhan University, Wuhan, China

**Keywords:** clear cell renal cell carcinoma, methylation CpG island, prognostication panel, elastic net, survival analysis

## Abstract

DNA methylation based prognostic factor for patients with clear cell renal cell carcinoma (ccRCC) remains unclear. In the present study, we identified survival-related DNA methylation sites based on the differentially methylated DNA CpG sites between normal renal tissue and ccRCC. Then, these survival-related DNA methylation sites were included into an elastic net regularized Cox proportional hazards regression (CoxPH) model to build a DNA methylation-based panel, which could stratify patients into different survival groups with excellent accuracies in the training set and test set. External validation suggested that the DNA methylation-based panel could effectively distinguish normal controls from tumor samples and classify patients into metastasis group and non-metastasis group. The nomogram containing DNA methylation-based panel was reliable in clinical settings. Higher total mutation number, SCNA level, and MATH score were associated with higher methylation risk. The innate immune, ratio between CD8^+^T cell versus Treg cell as well as Th17 cell versus Th2 cell were significantly decreased in high methylation risk group. In inclusion, we developed a DNA methylation-based panel which might be independent prognostic factor in ccRCC. Patients with higher methylation risk were associated genomic alteration and poor immune microenvironment.

## Introduction

Renal cancer, also known as renal cell carcinoma (RCC), renal adenocarcinoma, etc., accounts for about 85% of primary renal malignancies (Capitanio and Montorsi, 2016; Morris and Latif, 2017). Nearly 70–80% of RCCs are defined as clear cell renal cell carcinoma (ccRCC) (Capitanio and Montorsi, 2016). The cause of kidney cancer has not yet been clarified, and its onset may be related to smoking, obesity, occupational exposure (such as asbestos, leather, etc.), and genetic factors (such as the absence of tumor suppressor genes) (Capitanio and Montorsi, 2016). About 30–50% of kidney cancer lacks early clinical manifestations, while nearly two-thirds of ccRCCs are detected incidentally (Gonzalez Leon and Morera Perez, 2016; Hancock and Georgiades, 2016). It has been reported that nearly 30–50% of ccRCCs will develop metastases even after undergoing radical surgery during follow-up (Fidler, 1992; Bolger, 2002; Singh et al., 2017).

Tumor stage, nuclear grade, morphology, and histology determined at the time of nephrectomy have traditionally been applied to prognosticate the progression and survival of patients with ccRCC (Golovastova et al., 2017). However, recent studies suggested that molecular heterogeneity was involved in the carcinogenesis and development of ccRCC tumors, leading to variation in cellular proliferation, metabolic activity and tumor microenvironment of ccRCC (Borys et al., 2019; Liss et al., 2019). Thus, the above traditionally clinicopathological features might be insufficient for precise tumor characterization and classification and survival prognostication (Moch et al., 2014; Zheng et al., 2018).

Currently, thanks to high-throughput sequencing technology, a variety of novel biomarkers have been introduced in clinical settings (van Vlodrop et al., 2017; Liu et al., 2018; Wu et al., 2019). Epigenetic alterations have been demonstrated to serve as key contributors to the regulation of gene expression and involved in various kinds of malignancies as well as the clinicopathology of other medical conditions (Morris and Latif, 2017; Young et al., 2017; Evelonn et al., 2019). Global DNA hypomethylation and regional hypermethylation of Cytosine-Phosphate-Guanine (CpG)-rich islands are identified in multiple kinds of malignancies, and are associated with gene expression and interact with various positive and negative feedback mechanisms (de Cubas and Rathmell, 2018). Therefore, aberrant methylation CpG sites could be selected as novel prognostication markers in human cancers including ccRCC.

In the present study, we identified a combination of 11 methylation sites associated with the survival, genomic alteration, and tumor microenvironment of patients with ccRCC, and suggested that it could be an independent prognostic factor in patients with ccRCC.

## Materials and Methods

### Patient Cohort

A total of 483 renal samples in the TCGA Kidney Clear Cell Carcinoma (TCGA-KIRC) cohort obtained at the time of nephrectomy were subjected to the Illumina Infinium 450k Human DNA methylation Beadchip to obtain genome wide DNA methylation status in approximately 485,000 CpGs in renal carcinoma and adjacent normal kidney tissue samples from ccRCC patients (Cancer Genome Atlas Research Network, 2013). We obtained the beta value matrix of the TCGA-KIRC methylation profile from UCSC Xena^1^. Meanwhile, we obtained associated copy number data, mRNA expression data, somatic mutation data and clinical data of TCGA-KIRC from GDC data portal^2^. GSE113501, measured by Illumina HumanMethylation450 BeadChip and included 115 ccRCC patients and the associated methylation profile, were downloaded for Gene Expression Omnibus (GEO)^3^ (Evelonn et al., 2019). We used GSE113501 as an independent validation set to verify the relationship between methylation status and disease metastasis. GSE61441, measured by Illumina HumanMethylation450 BeadChip, included 46 ccRCC tissue and 46 matched normal kidney tissues, were used to validate the relationship between the methylation status and ccRCC and normal kidney tissue (Wei et al., 2015).

### Differential Methylation Analysis

R/Bioconductor package ChAMP was used to identify differentially methylated CpG sites (Morris et al., 2014). Before performing differential methylation analysis, probes met the following criteria were filtered: (1) non-CpG probes; (2) probes with SNPs; (3) probes aligning to multiple location; (4) probes located on the X, Y chromosome. Then, CpG sites with adjusted *P* < 0.05 and cut.deltaBeta > 0.3 were considered to be significantly differentially methylated.

### Identification of Prognosis-Related CpG Sites and Development of DNA Methylation-Based Panel

The TCGA-KIRC cohort was randomly partitioned into two analyzing groups - training set and test set, in a ratio of 3:2. On the basis of the above differentially methylation CpG sites, we tried to identify prognosis-related CpG sites that were significantly (sites with Bonferroni adjusted *P* less than 0.01) associated with the overall survival (OS) of patients with ccRCC using univariate Cox proportional hazards regression model (CoxPH). Then, the prognosis-related CpG sites were included in an elastic net regularized CoxPH model (Zou and Hastie, 2005). The two hyperparameters α and λ were tuned through 10-fold cross-validation using the R package “glmnet” (Simon et al., 2011) and “c060” (Sill et al., 2014). CpG sites with non-zero coefficients in the elastic net regularized CoxPH model were integrated to build a lineal risk score based on the methylation levels of the CpG sites and their corresponding coefficients in the model.

### Characterizing the Prognostication Ability of the DNA Methylation-Based Panel

Univariate and multivariable CoxPH model were performed to analyze the relation between the DNA methylation-based panel and the OS of patients with ccRCC in clinical settings. For the CoxPH model, the DNA methylation-based panel, age, and tumor stage were continuous variables, while hemoglobin, serum calcium, and gender were taken as categorized variable. Time-dependent receiver operating characteristic curve (ROC) analysis were performed using the R package “survivalROC” (Kamarudin et al., 2017), and the 1-, 3-, 5-, 7-, and 10-year Area under the ROC Curves (AUCs) were visualized using the R packages “ggplot2.” Moreover, the prediction ability of the DNA methylation-based panel was also validated in two independent validation cohorts (GSE61441, Wei et al., 2015; and GSE113501, Evelonn et al., 2019). The risk scores of patients in the two cohorts were calculated based on the methylation levels of the CpG sites in the DNA methylation-based panel and their responding coefficients in the elastic net penalized CoxPH model.

### Clinical Application of the DNA Methylation-Based Panel

To determine the clinical utility of the methylation-based panel, the DNA methylation-based panel and other clinical variables (age, hemoglobin level, serum calcium level, tumor stage, and gender) were incorporated into a multivariable survival model, and then developed a nomogram incorporating the DNA methylation-based panel. The nomogram was also calibrated at 3-, and 5- years. Moreover, decision curve analysis (DCA) (Vickers and Elkin, 2006) was performed to determine the clinical benefit of the DNA methylation containing panel. Finally, we compared the prognostic performance of the DNA methylation-based panel with those of several currently available biomarkers in the whole TCGA-KIRC cohort using C-index.

### Correlation Analysis Between the CpG Site Methylation and the Corresponding Gene Expression

The fragments per kilobase of exon model per million reads mapped (FPKM) normalized gene expression profile of the TCGA-KIRC cohort was used to identify the relationship between the CpG site methylation status and the expression of genes regulated by the corresponding sites. To this end, Spearman’s correlation analysis was performed and visualized using the R package “ggpubr.” Meanwhile, the role of specific gene on the OS of patients with ccRCC was also characterized.

### Analyzing Associations Between the DNA Methylation-Based Panel and Genomic Metrics of Patients With ccRCC

To investigate the mechanisms that the DNA methylation-based panel affected the survival of ccRCC patients, we performed Spearman’s rank correlation analysis to characterize the relationship between the methylation risk of ccRCC patients and genomic metrics of ccRCC patients including total numbers of mutations, clonal heterogeneity measured by the mutant-allele tumor heterogeneity (MATH) (Mroz and Rocco, 2013), and somatic copy number alteration (SCNAs) (Mroz and Rocco, 2013). R package “maftool” (Mayakonda et al., 2018) was used to calculate the total mutation number and MATH of each ccRCC on the basis of VarScan2 preprocessed mutation data. The SCNA levels of ccRCC patients in the TCGA-KIRC cohort were calculated as previously introduced (Davoli et al., 2017).

### Analyzing the Associations Between the DNA Methylation-Based Panel and Tumor Microenvironment (TME) of Patients With ccRCC

Next, we tried to study the relationship between the methylation status of the multi-CpG site and tumor microenvironment. We used gene signatures of 24 types of immune cells introduced by Bindea et al. (2013) to calculate the specific immune infiltration using the single cell gene set enrichment analysis (ssGSEA) method implemented in the R package GSVA (Hanzelmann et al., 2013). As introduced by Senbabaoglu et al. (2016), we used the mean of the standardized values for CD8^+^ T cell, T helper cell, T cell, T central and effector memory cell, Th1 cell, Th2 cell, Th17 cell, and Treg cells as T cell infiltration score (TIS), while the mean standardized value of the 24 types of immune cells was treated as overall immune infiltration score (OIIS). Moreover, we analyzed the correlation between the methylation risk and four inhibitory checkpoint molecules (PDCD1, PDCD1LG2, CD274, and CTLA-4). Spearman’s rank correlation analysis was used to characterize the correlations between specific immune cell composition (or checkpoint molecules) and the methylation risk of the multi-CpG site.

### Detection of the Methylation Levels of the Multi-CpG Sites in Clinical Settings

ccRCC samples and corresponding normal renal tissues were collected from patients who received surgery during hospitalization at department of urology, Zhongnan hospital of Wuhan university, which was approved by the Ethics Committee at Zhongnan Hospital of Wuhan University, the informed consent was available from all participants as well. The procedure of bisulfite sequencing PCR (BSP) and methylation specific PCR (MSP) and data analysis were the same with our previous study (Ge et al., 2019).

## Results

### Differentially Methylated CpG Sites Between ccRCC and Adjacent Normal Renal Tissue

**FIGURE 1:**
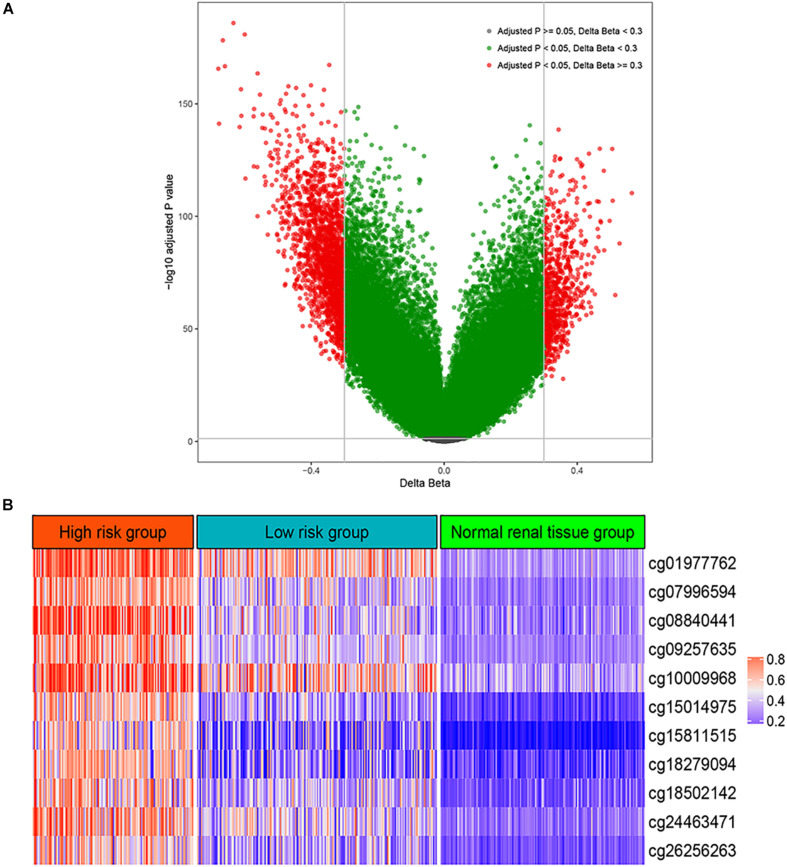
Differential methylation analysis. **(A)** Volcano plot for differentially methylated CpG sites between normal renal tissue and renal cell carcinoma. **(B)** The methylation levels of the 11 CpG site in the high risk group, low risk group, and normal renal tissue group.

A total of 483 renal tissues were subjected to DNA methylation profiling, including 160 pairs of ccRCC sample and adjacent normal renal samples. Thus, we calculated differentially methylated CpG sites based on the paired tumors and normal samples. As shown in Figure 1A, a total of 2,628 CpG sites were found to be differentially methylated between ccRCC and normal renal tissue at adjusted *P* < 0.05 and cut.deltaBeta > 0.3.

### Development of a Combination of Multi-CpG Site (DNA Methylation-Based Panel) Relating With the Survivals of Patients With ccRCC

Firstly, a total of 56 CpG sites were shown to be significantly (Bonferroni adjusted *P* < 0.01) correlated with the overall survival (OS) of patients with ccRCC using univariate CoxPH model (Supplementary Table S1). Then, these 56 CpG sites were included into an elastic net penalized CoxPH model. After 10-fold cross-validation, optimal α (0.0129) and λ (10.6629) were utilized to fit a final elastic net penalized CoxPH model, resulting in 11 CpG sites whose coefficients were not zero (Supplementary Figure S1 and Supplementary Table S1). Therefore, we built a DNA methylation-based panel based on the coefficients and methylation levels of these 11 CpG sites (Figure 1B and Supplementary Figure S2).

### Prognostication Relevance of the DNA Methylation-Based Panel

**FIGURE 2:**
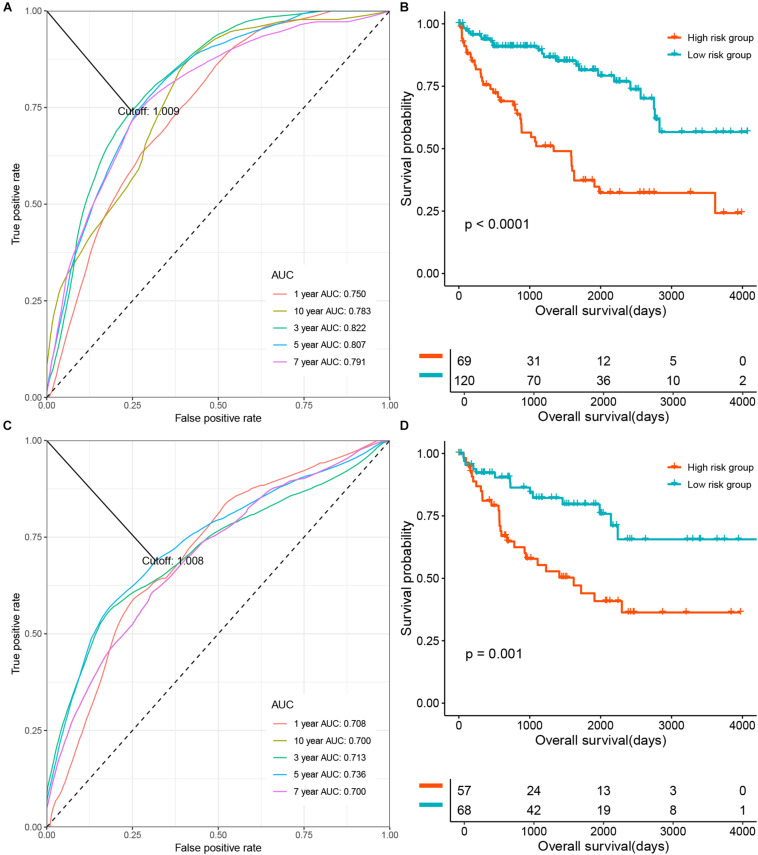
The prognosis relevance of the DNA methylation panel. **(A)** Time dependent Receiver operating characteristic (ROC) analysis of the prediction ability at the DNA methylation panel at different time points in the training set. **(B)** The overall survival (OS) of patients in the methylation low risk group and the high risk group in the training set. **(C)** ROC analysis of the prediction ability at the DNA methylation panel at different time points in the test set. **(D)** The OS of patients in the methylation low risk group and the high risk group in the test set.

In order to determine the prognostication relevance of the DNA methylation-based panel, patients were categorized into two risk groups according to the optimal cutoff of the risk score calculated based on the coefficients and methylation levels of these 11 CpG sites. Then, we performed survival analysis on the OS of ccRCC patients. As shown in Figure 2A, in the training set, time-dependent receiver operating characteristic (ROC) analysis suggested that the area under the ROC curves (AUCs) at 1-, 3-, 5-, 7-, and 10-year were 0.750, 0.783, 0.822, 0.807, and 0.791, respectively. Kaplan-Meier (KM) curve suggested patients in the low-risk group demonstrated significantly longer OS compared with those in the high-risk group (*P* < 0.0001) (Figure 2B). Meanwhile, time-dependent ROC analysis also suggested that the DNA methylation-based panel showed high sensitivity and specificity in predicting the OS of ccRCC patients in the test set, with 1-, 3-, 5-, 7- and 10-year AUCs of 0.708, 0.700, 0.713, 0.736, and 0.700, respectively (Figure 2C). KM survival analysis suggested that the DNA methylation-based panel could classified patients into significant risk groups (*P* = 0.001) (Figure 2D). Moreover, multivariable CoxPH model suggested the DNA methylation-based panel was an independent prognostication factor after adjusting other clinical variables including age, hemoglobin level, serum calcium level, tumor stage, and gender (Supplementary Tables S2, S3).

### External Validation of the Prediction Ability of the DNA Methylation-Based Panel

**FIGURE 3:**
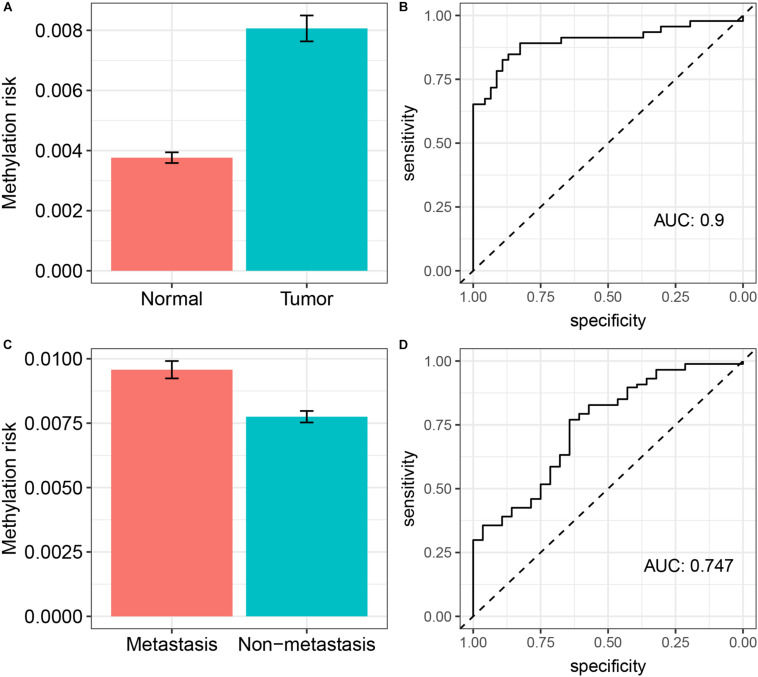
External validation of the prediction ability of the DNA methylation-based panel. **(A)** The methylation risk of patients in normal renal tissue group and ccRCC group and its diagnostic accuracy **(B)**; **(C)** the methylation risk of patients who develop metastasis and not develop metastasis and its diagnostic accuracy **(D)**.

Next, we tried to validate the prediction ability of the DNA methylation-based panel in two independent validation cohorts (GSE61441, Wei et al., 2015; GSE113501, Evelonn et al., 2019). We calculated the risk scores of the 46 paired ccRCC tumor and adjacent normal kidney tissues (GSE61441) as well as the risk scores of the ccRCC samples in GSE113501 on the basis of the methylation levels of the CpG sites in the DNA methylation-based panel and their responding coefficients in the elastic net penalized CoxPH model. As shown in Figures 3A,B, the risk scores of tumor samples (ccRCC) were significantly higher(Wilcox test *P* < 0.0001) compared with those of adjacent normal tissues, which achieved an AUC of 0.9. Meanwhile, the DNA methylation-based panel could also significantly predict the metastasis of ccRCC (Wilcox test *P* < 0.0001), with an AUC of 0.747 (Figures 3C,D).

### DNA Methylation-Based Panel Containing Nomogram and Its Clinical Application

**FIGURE 4:**
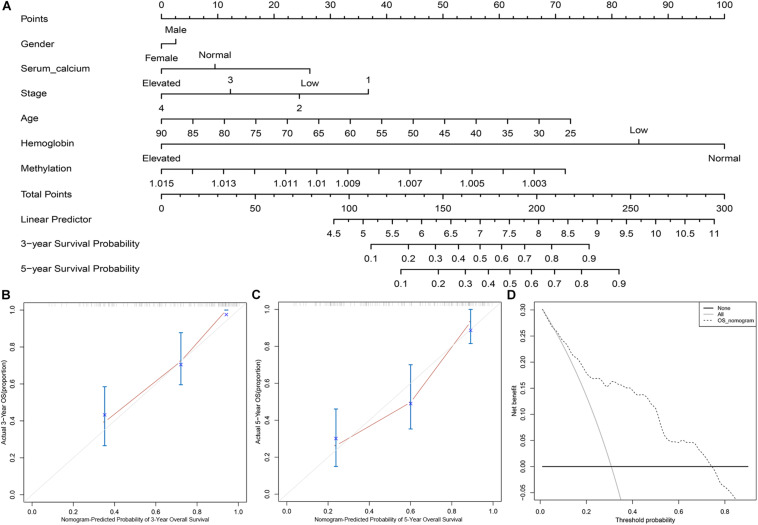
The clinical application of the DNA methylation panel containing nomogram. **(A)** Nomogram incorporating the DNA methylation-based panel, age, hemoglobin level, serum calcium level, tumor stage, and gender. **(B)** Calibration analysis of the prediction ability of the nomogram at 3 years. **(C)** Calibration analysis of the prediction ability of the nomogram at 5 years. **(D)** Decision curve analysis on the nomogram.

To determine the clinical utility of the methylation-based panel, we incorporated the DNA methylation-based panel and other clinical variables (age, hemoglobin level, serum calcium level, tumor stage, and gender) into a multivariable survival model, and then developed a nomogram incorporating the DNA methylation-based panel (Figure 4A). Clinically, the nomogram could be used in such a way that the score (according to the “Points” line) of each variable is calculated, and then the score of each variable is summed to obtain a total score (according to the “Total points” line), and then a physician could estimate the 3-year survival probability and 5-year survival probability of patients with ccRCC based on the total score of each patient. The C-indexes were 0.7447 for internal validation with bootstrap method and 0.788 for external validation, respectively. Meanwhile, calibration analysis suggested a good agreement between the predicted and observed 3-, and 5- year OS probabilities (Figures 4B,C). Moreover, DCA curve suggested that the DNA methylation panel containing nomogram showed better prediction ability for the OS within a threshold probability of 1–75% (Figure 4D).

### Comparison of Prognostication Performance Between the DNA Methylation-Based Panel and Other Existing Prognostic Markers

**FIGURE 5:**
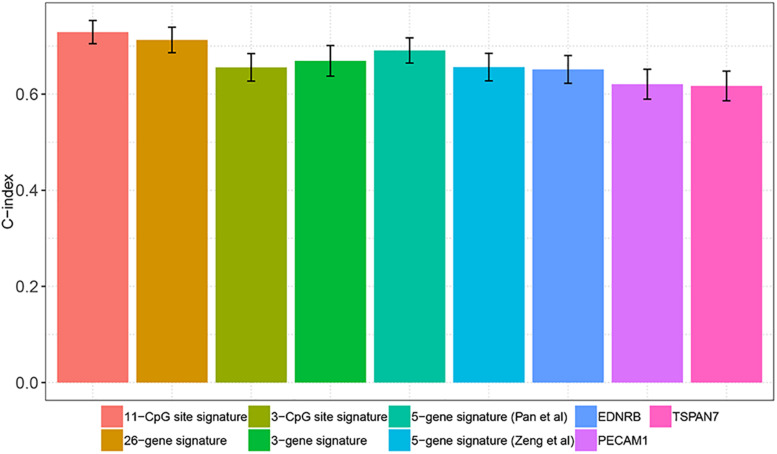
Comparison of prognostication performance between the DNA methylation panel and other existing prognostic markers.

Several studies have reported that a variety of molecular markers can be used to predict the survival of patients with ccRCC. Wuttig et al. (2012) suggested that PECAM1, EDNRB, and TSPAN7 could predict the survival of ccRCC patients. Kosari et al. (2005) classified ccRCC patients into aggressive and non-aggressive groups using a 36-gene signature and confirmed that this 35-gene signature predicts patient survival. Pan et al. (2019) introduced a 5-gene signature including OTX1, MATN4, PI3, ERVV-2, and NFE4 that was associated with the progression and prognosis of ccRCC patients. Kang et al. (2019) identified a 3- CpG site based promoter methylation signature associated with aggressive tumor phenotype and progression free survival in patients with ccRCC after surgical treatment. Zeng et al. (2019) indicated that the combination of IDUA, NDST1, SAP30L, CRYBA4, and SI was a prognostic signature (5-gene signature) in ccRCC. Chen et al. (2019) applied a least absolute shrinkage and selection operator (LASSO) penalized CoxPH model to identified a 3-gene signature that showed significantly prognostic ability in ccRCC. In the present study, we tried to evaluate and compare the prognostication performances of the above prognostic biomarkers and our DNA methylation-based panel based on C-index. As shown in Figure 5, the C-index of our DNA methylation-based panel was significantly higher compared with the 3-CpG site signature (0.73 versus 0.66, *P* = 0.0295), 3-gene signature (0.73 versus 0.67, *P* = 0.024), two 5-gene signatures (0.73 versus 0.66, *P* = 0.0114 and 0.73 versus 0.69, *P* = 0.0543), EDNRB (0.73 versus 0.65, *P* = 0.0015), TSPAN7 (0.73 versus 0.62, *P* = 0.0002), and PECAM1 (0.73 versus 0.62, *P* < 0.0001). At the same time, the predictive performance of our DNA methylation-based panel was comparable with the 35-gene signature (0.73 versus 0.71, *P* = 0.2777).

### Associations Between Methylation and Gene Expression and the Impact of the Associated Gene Expression on the Survival of Patients

As an epigenetic modification, DNA methylation has been demonstrated to play an important role in the regulation of gene expression (Spainhour et al., 2019). Thus, we tried to identify the correlations between the methylation levels of the 11 CpG sites and the expression levels of their corresponding genes. Unlike conventional understanding, the methylation levels in most sites are positively correlated with gene expressions. CpG site cg10009968 methylation level was positively correlated with the expression of CARD11 (*R* = 0.37, *P* < 0.0001); Methylation levels of cg07996594 (*R* = 0.43, *P* < 0.0001), cg15014975 (*R* = 0.39, *P* < 0.0001), cg24463471 (*R* = 0.43, *P* < 0.0001), and cg26256263 (*R* = 0.4, *P* < 0.0001) were positively correlated with the expression levels of RUNX3; The methylation level of CpG site cg01977762 was positively correlated with the expression level of UHRF1 (*R* = 0.32, *P* < 0.0001) and cg08840441 methylation was positively correlated with GMIP expression (*R* = 0.45, *P* < 0.0001); Meanwhile, there was a trend of positively correlation between the methylation level of CpG site cg15811515 (*R* = 0.075, *P* = 0.19) and the expression level of CSDAP1; Moreover, the expression of genes related with the 11 CpG sites could significantly stratified patients into different survival groups (Supplementary Figure S3).

### Associations Between Genomic Metrics and the Methylation Risk of ccRCC Patients

**FIGURE 6:**
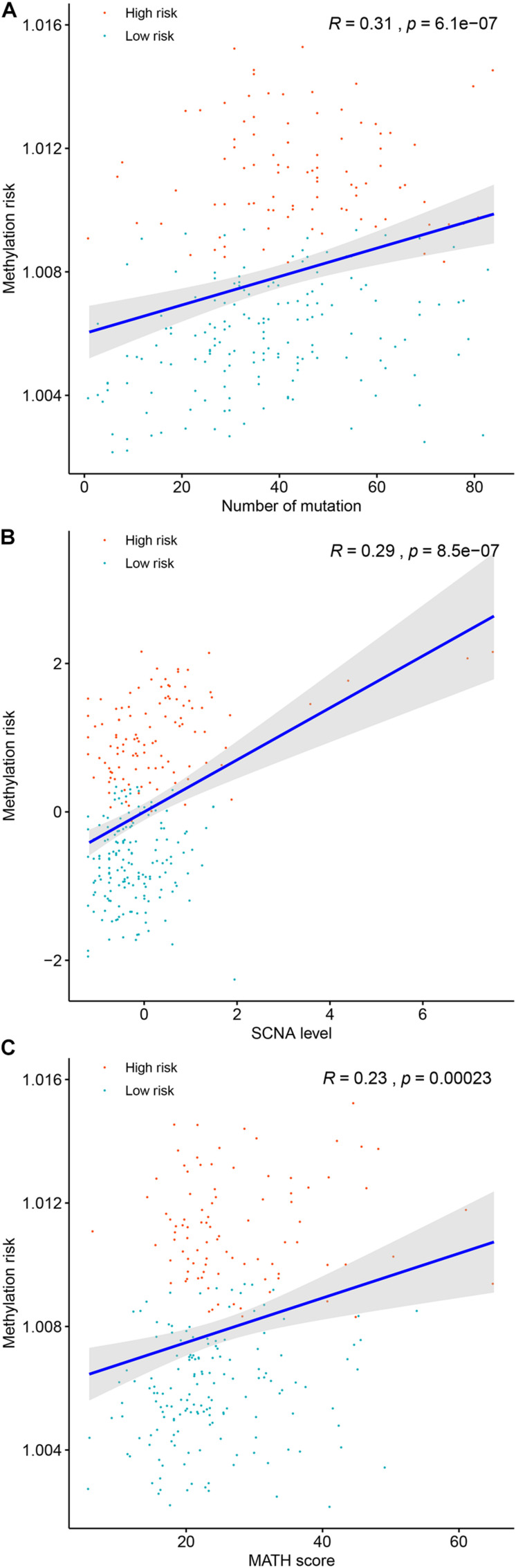
The associations between the DNA methylation risk and total mutation number **(A)**, mutant-allele tumor heterogeneity **(B)**, and somatic copy number alterations **(C)**.

Genomic abnormalities have been found in a variety of human tumors and global genomic aberration was found in the progression serval human malignance and closely related to their progression. Thus, we analyzed the correlations between common genomic abnormalities (total mutation load, SCNA and MATH and the DNA methylation-based panel associated methylation risk of ccRCC patients). As shown in Figure 6, the methylation risk of ccRCC patients was significantly correlated with total mutation number (*R* = 0.31, *P* < 0.0001, Figure 6A), SCNA (*R* = 0.29, *P* < 0.0001, Figure 6B), and MATH (*R* = 0.23, *P* < 0.0001, Figure 6C). These findings indicated that patients with higher methylation risk were associated with greater clonal heterogeneity.

### Association Between Immune Cell Infiltration and the Methylation Risk of ccRCC Patients

**FIGURE 7:**
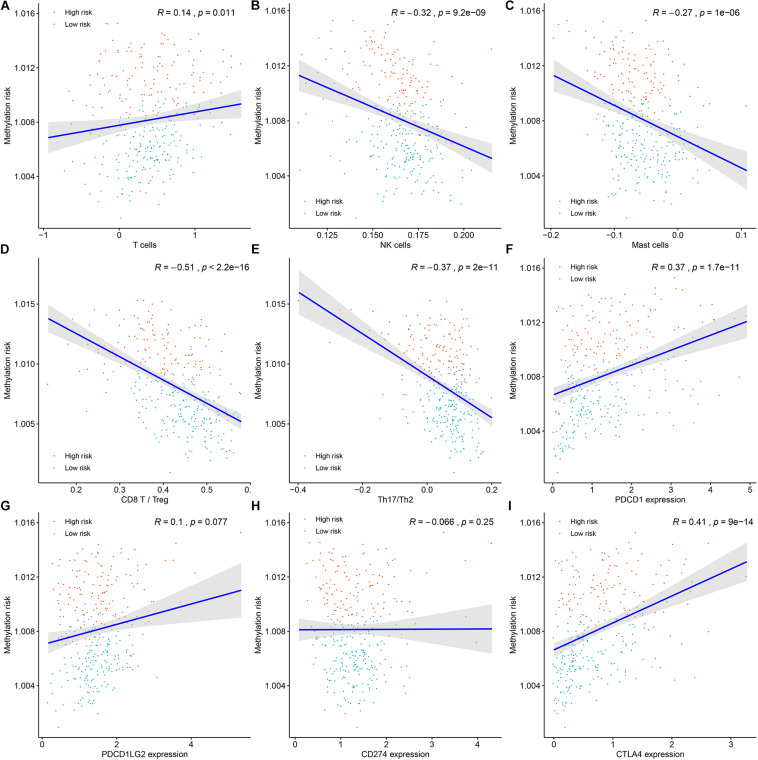
The associations between the DNA methylation risk and the tumor microenvironment (TME) of patients, including T cell infiltration score **(A)**, NK cells **(B)**, mast cells **(C)**, the ratio between CD8^+^ T cells versus Treg cells **(D)**, the ratio between Th17 cells versus Th2 cells **(E)**, the expression of PDCD1 **(F)**, the expression of PDCD1LG2 **(G)**, the expression CD274 **(H)**, and the expression of CTLA4 **(I)**.

Tumors are not just masses of monoclonal malignant cells, it also encompasses a variety of immune cells and stromal cells, which together constitute a complex tumor microenvironment. Tumor-infiltrating immune cells have been reported to play a significant role in tumor progression and management. Therefore, determination of the relationship between the methylation risk and the tumor-infiltrating immune cells could help clarify the mechanisms underlying the survival impact of the methylation of the 11 CpG sites and help design immunogenic effects of anticancer therapies. ssGSEA-based immune cell infiltration level was calculated as mentioned above. As shown in Figure 7A and Supplementary Figure S4, Spearman’s rank correlation analysis revealed weak positive associations between the methylation risk and T cell infiltration score (TIS) (*R* = 0.14, *P* = 0.011) and overall immune infiltration score (OIIS) (*R* = 0.15, *P* = 0.011). While significantly negatively correlations were found between the methylation risk and innate immune cells of NK cells (*R* = −0.32, *P* < 0.0001, Figure 7B) and mast cells (*R* = −0.27, *P* < 0.0001, Figure 7C).

The ratio between protumorigenic immune cells versus antitumorigenic cells is more likely to determine whether the net effect of these cells is tumor promotion versus inhibition compared with the absolute count of certain immune cell types. As shown in Figures 7D,E, the ratio between CD8^+^ T cells versus Treg cells (*R* = −0.51, *P* < 0.0001) as well as the ratio between Th17 cells versus Th2 cells (*R* = −0.31, *P* < 0.0001) was negatively correlated with the methylation risk of ccRCC patients. Next, we tried to determine the associations between the expression of immunotherapeutic targets PD-1 (PDCD1), PD-L1 (CD274), PDCD1LG2 and CTLA-4 (CTLA4) (Figures 7F–I)and the methylation risk of ccRCC patients, and Spearman’s correlation analysis revealed that the expressions of PDCD1 (*R* = 0.37, *P* < 0.0001, Figure 7F), PDCD1LG2 (*R* = 0.1, *P* = 0.077, Figure 7G), and CTLA4 (*R* = 0.41, *P* < 0.0001, Figure 7I) were positively correlated with the methylation risk of patients with ccRCC.

### The Methylation Levels of the CpG Sites in ccRCC Samples and Its Normal Controls

We investigated the methylation levels of our the 11 CpG sites in 5 ccRCCs and 5 normal controls. As shown in Supplementary Figure S5, the methylation levels of cg15014975, cg07996594, cg26256263, cg18502142, cg15811515, cg09257635, cg10009968, cg08840441, and cg01977762 was obviously higher in ccRCCs compared with those in normal renal tissues (no significantly differences regarding the methylation levels of the others two CpG sites (cg24463471 and cg18279094) between the two groups), which was in accordance with the result of differentially methylation analysis above. Therefore, these results indicated that the 11 CpG sites containing DNA methylation panel would be reliable in clinical settings.

## Discussion

In the present study, a total of 2,628 of 361,127 CpG sited were differentially methylated between normal renal tissue and ccRCC and the global picture of DNA methylation patients in the high risk group, low risk group, and normal renal tissue group was shown in Supplementary Figure S6. Furthermore, we randomly divided patients in the TCGA-KIRC cohort into a training set and a test set, and used the elastic net regularized CoxPH model in the training set to find 11 methylation sites closely related to the overall survival of the patient. At the same time, we used the univariate and multivariable CoxPH models in the training set and test set to verify the predictive performance of the DNA methylation-based panel on the OS of ccRCC patients.

In clinical applications, we constructed a nomogram containing the DNA methylation-based panel and other clinical features such as patient age, gender, and pathological stage, etc., and confirmed the reliability of its clinical use using internal and external validation methods. External validation results indicated that the DNA methylation-based panel was able to significantly distinguish between normal renal tissue and ccRCC as well as metastasis and non-metastasis ccRCCs. Finally, in predicting the OS of ccRCC patients, we confirmed that our DNA methylation-based panel was superior to other existing predictive molecular markers. Taken together, the above results indicated that the DNA methylation-based panel exhibited strong predictive value and might serve as an independent prognostic factor in patients with ccRCC.

Of the 11 CpG sites found in this study, 8 CpG sites (cg10009968, cg15811515, cg18279094, cg07996594, cg15014975, cg24463471, cg26256263, and cg01977762) are located in the promoter regions of the corresponding genes, and there are 3 CpG sites (cg09257635, cg08840441, and cg18502142) located in the gene body region of the corresponding genes. It is generally believed that methylation modifications of the promoter region can down-regulate the expression of the corresponding genes. However, unlike traditional understanding, our correlation analysis indicated that the methylation modifications of some promoter regions were positively correlated with the expression of the corresponding genes. This was consistent with the findings of Spainhour et al. (2019), indicating that a large number of positive correlations (about 30%) between methylation and gene expression in the 33 cancer types of TCGA database. Thus, there is an amount of more to be learned regarding the role of DNA methylation beyond the commonly accepted silencing role.

Associations between genomic metrics and prognosis of patients with malignance have been reported previously. Thus, in the present study, we tried to identify the associations between commonly accepted genomic metrics (total mutation load, SCNA, and MATH) and the methylation risk of ccRCC patients. As expected, patients with higher methylation risk were associated with higher total mutation load, which was consistent with previous observations in other tumors. SCNA, also known as aneuploidy, was widespread in multiple cancers and had been recognized as a driver event in carcinogenesis (Davoli et al., 2017). It was widely accepted that higher SCNA levels were correlated with high proliferation of cancer cells and poorer survival of cancer patients (Davoli et al., 2017). Our correlation analysis suggested that higher SCNA levels of ccRCC patients were associated with higher methylation risk, which were associated with poor survival of ccRCC patients. As another genomic alteration, MATH, a measure of intratumor heterogeneity, utilized the broadness of the distribution of mutant allele frequencies and had been used to assess the clonal and genetic heterogeneity of tumors. Higher MATH score corresponded to higher heterogeneity which fosters tumor evolution (McGranahan and Swanton, 2017). In the present study, we showed that patients with higher MATH score were significantly associated higher methylation risk, which ultimately resulted in poor survivals of patients.

Tumor immune cell infiltration represented an important component of tumor microenvironment and was used to classified multiple tumors into different immune subtypes associated different heterogeneity and survival (Thorsson et al., 2018). In the present study, we investigated the associations between the methylation risk and the infiltrating immune cells of ccRCC patients. Higher innate immune activity (measured by NK cell score and mast cell score) was associated lower methylation risk, unexpectedly, we observed weak positive correlations between TIS (*R* = 0.14, *P* = 0.011) and OIIS (*R* = 0.15, *P* = 0.011) and the methylation risk of ccRCC patients. Given that functions of tumor-infiltrating immune cells are different. For example, CD8^+^ T cells are always associated with cytolytic activity, while regulatory T cells (Tregs) and tumor associated macrophages (TAMs) have been demonstrated to be correlated with pro-tumor functions (Bindea et al., 2013). Thus, we investigated the correlation between the ratio of protumorigenic immune cells versus antitumorigenic cells and the methylation risk of ccRCC patients. As a result, ratio between CD8^+^ T cells (antitumorigenic) versus Treg cells (protumorigenic) was significantly correlated with lower methylation risk of ccRCC patients. Meanwhile, we found the ratio between Th17 cells (antitumorigenic) versus Th2 (protumorigenic) cells was significantly decreased in high versus low methylation risk patients. PD-1, also known as PDCD1, is a member of the CD28 superfamily that negatively regulates T cell activation in immune responses and plays a crucial role in immune tolerance by delivering negative signals upon interaction with its two ligands, PD-L1(CD274) or PD-L2 (PDCD1LG2) (Jin et al., 2011). CTLA-4 (CTLA4) is another immune receptor that negatively regulated T cells activation in immune response (Egen et al., 2002). Through correlation analysis, we observed that higher methylation risk was correlated with higher expression of these immune inhabitation molecules (PD-1, PDCD1LG2, CTLA-4). This might explain why higher TIS and OIIS was associated with higher methylation risk, i.e., the increased expression of PD-1, PDCD1LG2, and CTLA-4 mediates immune tolerance.

Taken together, we developed a DNA methylation-based panel which might be an independent prognostic factor in ccRCC. High levels of total mutation number, SCNA level, and MATH score were associated with higher methylation risk. The innate immune, and ratios between CD8^+^T cell versus Treg cell as well as Th17 cell versus Th2 cell were significantly decreased in high methylation risk group.

## Data Availability Statement

Publicly available datasets were analyzed in this study. This data can be found here: TCGA-KIRC methylation profile was downloaded from UCSC Xena (https://xenabrowser.net/); copy number data, mRNA expression data, somatic mutation data and clinical data of TCGA-KIRC were downloaded from GDC data portal (https://portal.gdc.cancer.gov). GSE11501 were downloaded from https://www.ncbi.nlm.nih.gov/geo/query/acc.cgi?acc=GSE113501; GSE61441 were downloaded from https://www.ncbi.nlm.nih.gov/geo/query/acc.cgi?acc=GSE61441.

## Ethics Statement

The studies involving human participants were reviewed and approved by Ethics Committee at Zhongnan Hospital of Wuhan University. The patients/participants provided their written informed consent to participate in this study.

## Author Contributions

XW and SL designed the study. X-PL collected the data, performed the statistical analysis, conducted the methylation detection, and wrote the manuscript. LJ participated in the methylation detection. CC and TL reviewed the manuscript. All the authors contributed to the article and approved the submitted version.

## Conflict of Interest

The authors declare that the research was conducted in the absence of any commercial or financial relationships that could be construed as a potential conflict of interest.

## Footnotes

1 https://xenabrowser.net/

2 https://portal.gdc.cancer.gov

3 https://www.ncbi.nlm.nih.gov/geo/

## Supplementary Material

Supplementary Figure 1 Optimal α and λ tuned through 10-fold cross validation.

Supplementary Figure 2 The methylation differences of the 11 CpG site in the high risk group, low risk group, and normal renal tissue group.

Supplementary Figure 3 Associations between methylation and gene expression and the impact of the associated gene expression on the survival of patients.

Supplementary Figure 4 The associations between the DNA methylation risk and overall immune cell infiltration score.

Supplementary Figure 5 The relative methylation levels of the 11 CpG sites in five pairs of renal cell carcinoma and normal renal tissue. The relative methylation levels of cg15811515 in normal renal tissue were 0, thus, they are not presented in this figure.

Supplementary Figure 6 The global picture of DNA methylation patients in the high risk group, low risk group, and normal renal tissue group.

Supplementary Table 1 Characteristics of the included 11 methylated CpG sites.

Supplementary Table 2 Univariate and multivariable Cox proportional hazards regression model on the overall survival of patients in the training set.

Supplementary Table 3 Univariate and multivariable Cox proportional hazards regression model on the overall survival of patients in the testing set.
