# Supplementary material for: DNA Methylation-Based Panel Predicts Survival of Patients With Clear Cell Renal Cell Carcinoma and Its Correlations With Genomic Metrics and Tumor Immune Cell Infiltration
Source: Front Cell Dev Biol. 2020 Oct 15;8:572628. doi: 10.3389/fcell.2020.572628 (PMC7593608; doi:10.3389/fcell.2020.572628)
Supplement: Supplementary Figure 1 — Optimal α and λ tuned through 10-fold cross validation. [file Table_1.docx]

**Supplementary table 1** Characteristics of the included 11 methylated CpG sites

|  | Coefficient | HR | LCI | UCI | P Value | Adjusted P value | Gene symbol |  |  |  |  |
| --- | --- | --- | --- | --- | --- | --- | --- | --- | --- | --- | --- |
| cg01977762 | 3.162024 | 23.61834 | 7.086134 | 78.72078 | 2.63E-07 | 0.000692 | UHRF1 |  |  |  |  |
| cg07996594 | 2.775838 | 16.05208 | 6.060665 | 42.515 | 2.33E-08 | 6.12E-05 | RUNX3 |  |  |  |  |
| cg08840441 | 1.973876 | 7.198527 | 3.440183 | 15.0628 | 1.61E-07 | 0.000423 | GMIP |  |  |  |  |
| cg09257635 | 2.634073 | 13.93039 | 5.804281 | 33.43323 | 3.70E-09 | 9.73E-06 | GJC2 |  |  |  |  |
| cg10009968 | 2.383562 | 10.84346 | 4.052562 | 29.01389 | 2.07E-06 | 0.005436 | CARD11 |  |  |  |  |
| cg15014975 | 2.909641 | 18.35021 | 7.455717 | 45.16401 | 2.42E-10 | 6.37E-07 | RUNX3 |  |  |  |  |
| cg15811515 | 1.866766 | 6.467348 | 3.044541 | 13.73822 | 1.20E-06 | 0.003143 | CSDAP1 |  |  |  |  |
| cg18279094 | 2.559914 | 12.93471 | 5.856366 | 28.56835 | 2.42E-10 | 6.36E-07 | FOXD3 |  |  |  |  |
| cg18502142 | 2.183205 | 8.874702 | 3.871387 | 20.34422 | 2.50E-07 | 0.000656 | DLX6AS |  |  |  |  |
| cg24463471 | 2.522319 | 12.45745 | 5.079105 | 30.5542 | 3.58E-08 | 9.42E-05 | RUNX3 |  |  |  |  |
| cg26256263 | 2.586155 | 13.27862 | 5.679477 | 31.04541 | 2.40E-09 | 6.30E-06 | RUNX3 |  |  |  |  |

**Abbreviations:** HR, hazards ratio; LCI, lower limit of confidence interval; UCI, upper limit of confidence interval;

**Supplementary table 2** Univariate and multivariable Cox proportional hazards regression model on the overall survival of patients in the training set

| Characteristics | Univariate | | | |  | Multivariable | | | |
| --- | --- | --- | --- | --- | --- | --- | --- | --- | --- |
|  | HR | LCI | UCI | P value |  | HR | LCI | UCI | P value |
| Methylation risk | 4.8E+112 | 3.81E+75 | 6.1E+149 | 2.64E-09 |  | 1.11E+84 | 1.22E+33 | 1E+135 | 0.001227 |
| Age | 1.028987 | 1.008234 | 1.050167 | 0.005981 |  | 1.019388 | 0.990817 | 1.048782 | 0.185524 |
| Hemoglobin Elevated | Reference | | | |  | Reference | | | |
| Hemoglobin Low | 0.114567 | 0.014166 | 0.926569 | 0.042207 |  | 0.053542 | 0.005947 | 0.482071 | 0.009035 |
| Hemoglobin Normal | 0.044376 | 0.005184 | 0.379856 | 0.004461 |  | 0.031104 | 0.003393 | 0.28515 | 0.002141 |
| Serum calcium Elevated | Reference | | | |  | Reference | | | |
| Serum calcium Low | 0.282472 | 0.066197 | 1.205352 | 0.087698 |  | 0.536599 | 0.118466 | 2.430561 | 0.419281 |
| Serum calcium Normal | 0.382692 | 0.090389 | 1.620246 | 0.192047 |  | 0.703716 | 0.158062 | 3.133059 | 0.644684 |
| Tumor stage | 1.901602 | 1.52072 | 2.37788 | 1.74E-08 |  | 1.421422 | 1.062769 | 1.901111 | 0.017774 |
| Gender | 0.919856 | 0.543995 | 1.555411 | 0.755265 |  | 0.844914 | 0.45088 | 1.583301 | 0.598944 |

**Abbreviations**: HR, hazards ratio; LCI, lower limit of confidence interval; UCI, upper limit of confidence interval;

**Supplementary table 3** Univariate and multivariable Cox proportional hazards regression model on the overall survival of patients in the testing set

| Characteristics | Univariate | | | |  | Multivariable | | | |
| --- | --- | --- | --- | --- | --- | --- | --- | --- | --- |
|  | HR | LCI | UCI | P value |  | HR | LCI | UCI | P value |
| Methylation risk | 2.9E+87 | 4.27375E+47 | ## | 1.68E-05 |  | 8.523E+61 | 926.616279 | 8E+120 | 0.039534 |
| Age | 1.02924 | 1.000112406 | 1 | 0.04911 |  | 1.0269336 | 0.9845739 | 1.0711 | 0.216232 |
| Hemoglobin Elevated | Reference | | | |  | Reference | | | |
| Hemoglobin Low | 0.65192 | 0.153768963 | 3 | 0.561571 |  | 2.1736462 | 0.15317489 | 30.845 | 0.566186 |
| Hemoglobin Normal | 0.29041 | 0.064079768 | 1 | 0.108782 |  | 1.3109269 | 0.10821588 | 15.881 | 0.831536 |
| Serum calcium Elevated | Reference | | | |  | Reference | | | |
| Serum calcium Low | 0.03474 | 0.007830335 | 0 | 9.89E-06 |  | 0.0417591 | 0.00418535 | 0.4166 | 0.006811 |
| Serum calcium Normal | 0.0331 | 0.007158742 | 0 | 1.29E-05 |  | 0.0326612 | 0.00341629 | 0.3123 | 0.002974 |
| Tumor stage | 2.30666 | 1.7306175 | 3 | 1.19E-08 |  | 2.1913462 | 1.44918949 | 3.3136 | 0.0002 |
| Gender | 1.20227 | 0.639226552 | 2 | 0.567633 |  | 1.0802118 | 0.44407064 | 2.6276 | 0.864915 |

**Abbreviations:** HR, hazards ratio; LCI, lower limit of confidence interval; UCI, upper limit of confidence interval;


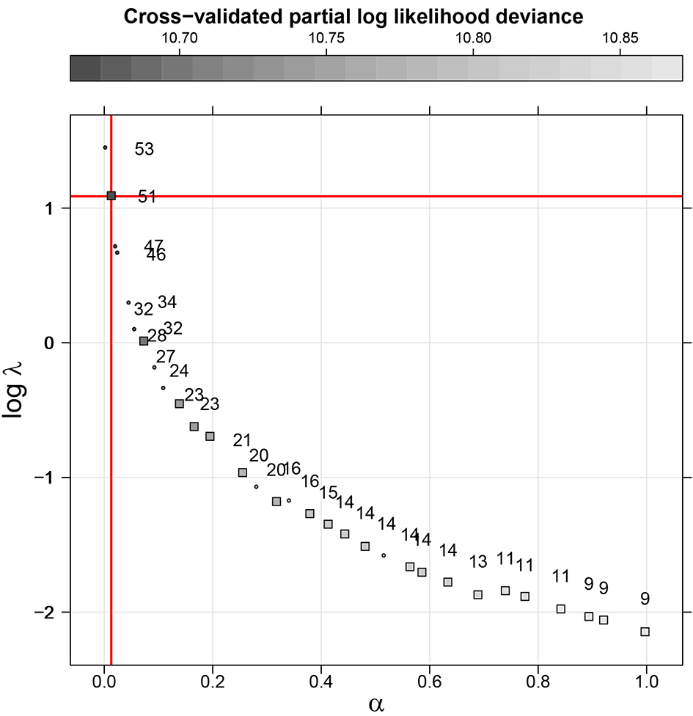


**Supplementary figure 1** Optimal α and λ tuned through 10-fold cross validation


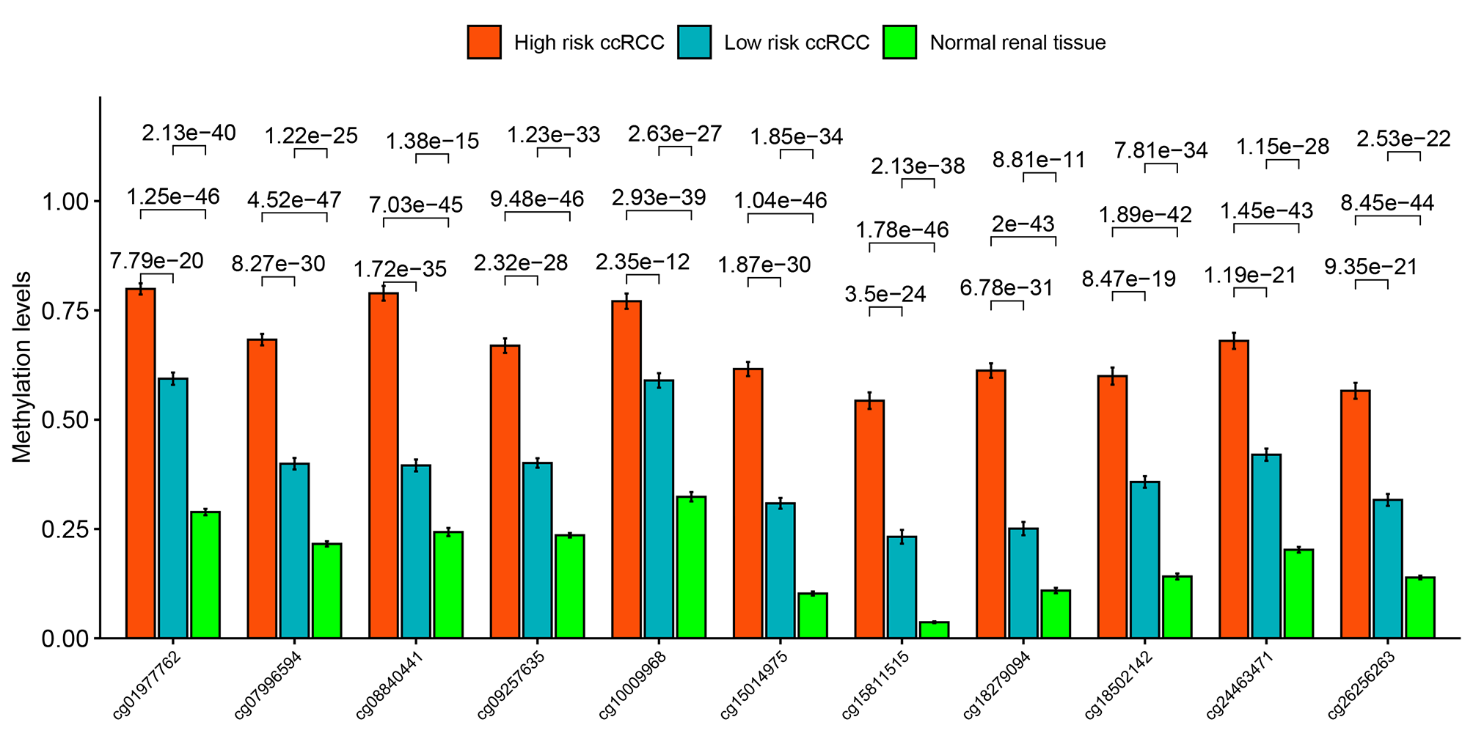


**Supplementary figure 2** The methylation differences of the 11 CpG site in the high risk group, low risk group, and normal renal tissue group


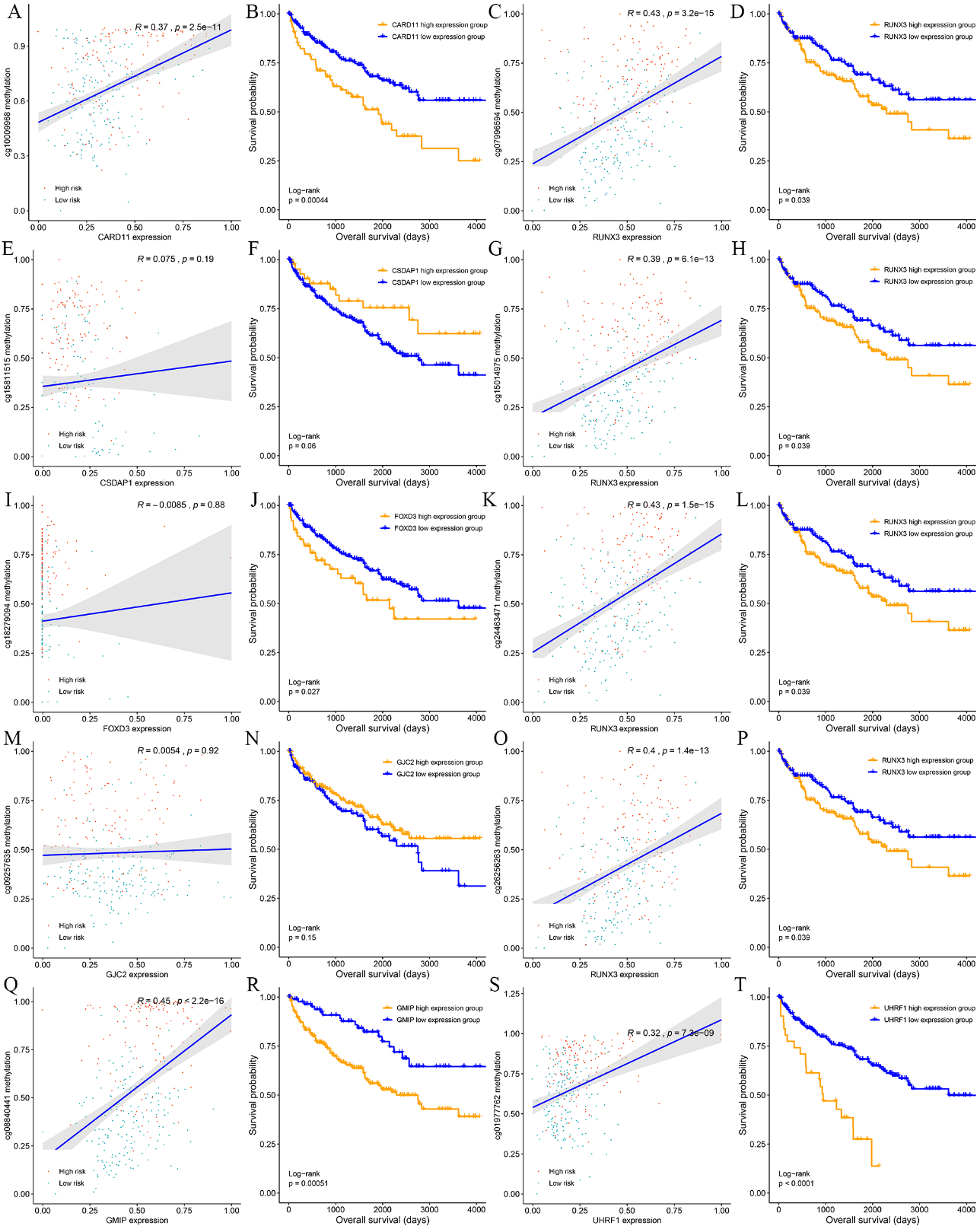


**Supplementary figure 3** Associations between methylation and gene expression and the impact of the associated gene expression on the survival of patients


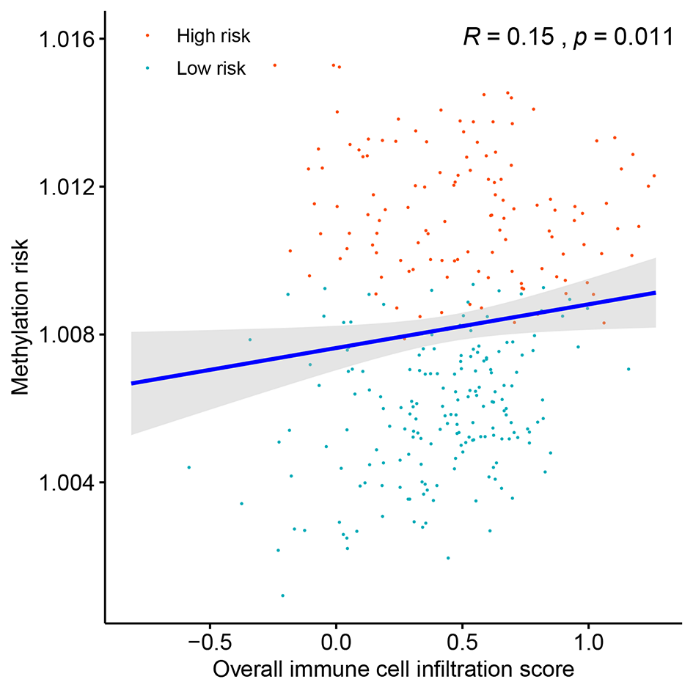


**Supplementary figure 4** The associations between the DNA methylation risk and overall immune cell infiltration score


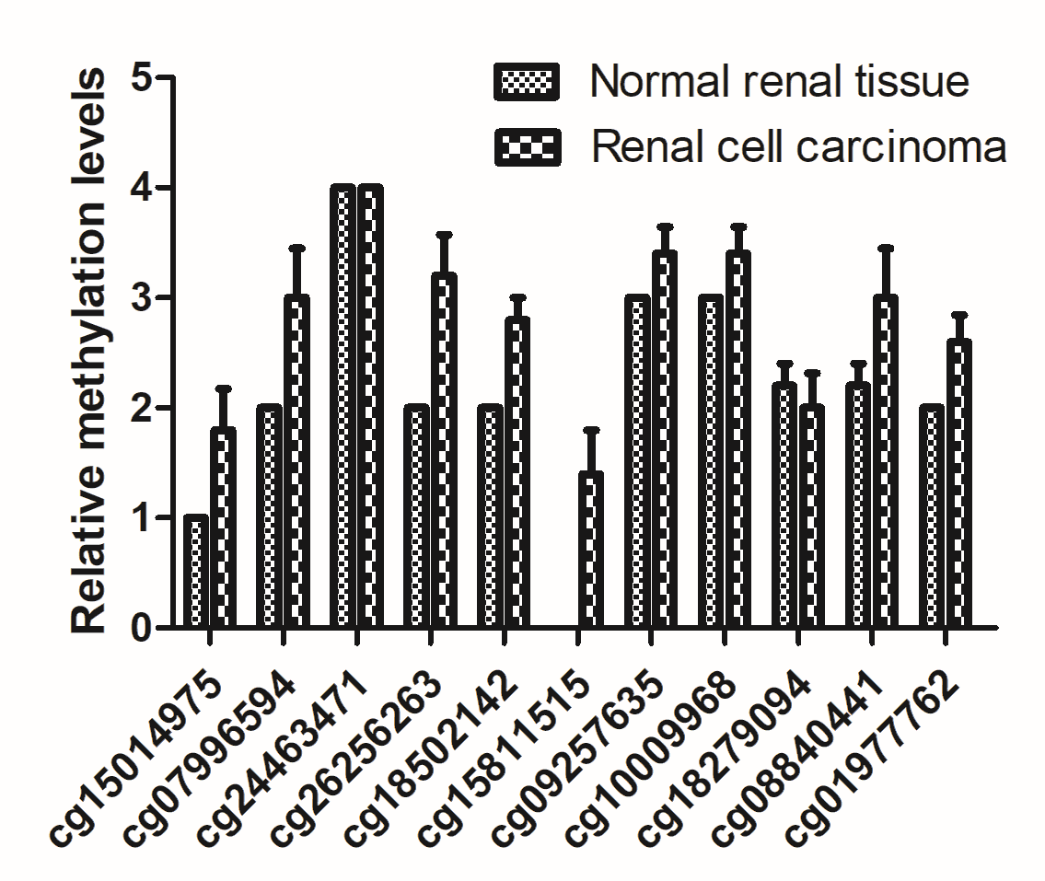


**Supplementary figure 5** The relative methylation levels of the 11 CpG sites in five pairs of renal cell carcinoma and normal renal tissue. Note: the relative methylation levels of cg15811515 in normal renal tissue were 0, thus, they are not presented in this figure.


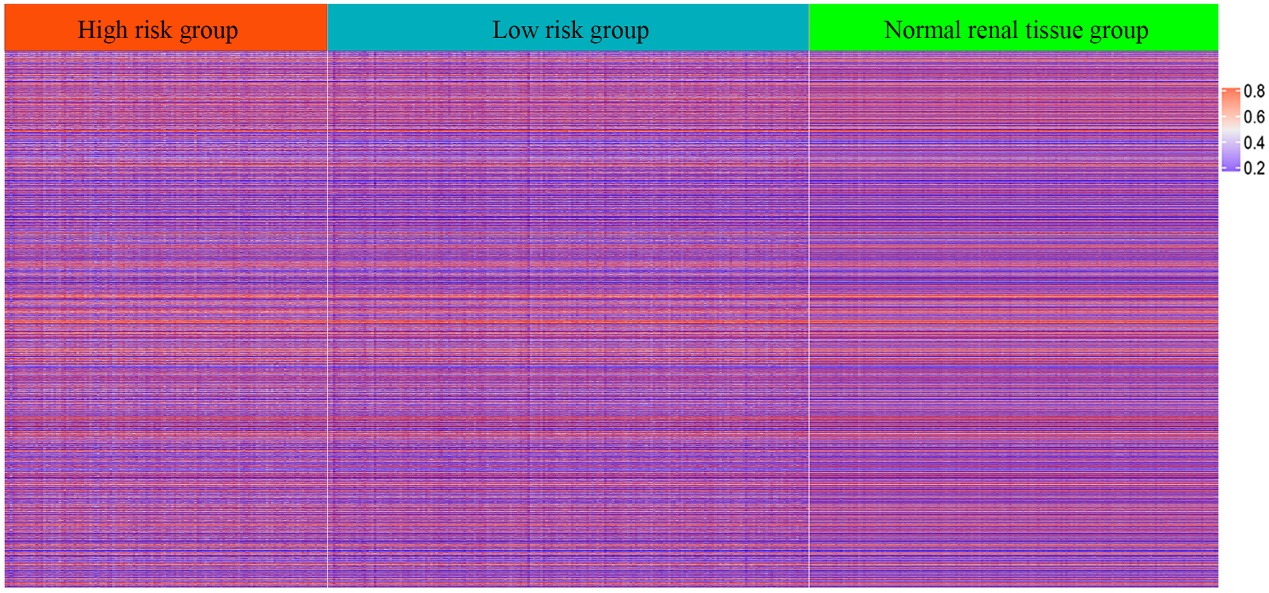


**Supplementary figure 6** The global picture of DNA methylation patients in the high risk group, low risk group, and normal renal tissue group
